# *Haloferax volcanii* N-Glycosylation: Delineating the Pathway of dTDP-rhamnose Biosynthesis

**DOI:** 10.1371/journal.pone.0097441

**Published:** 2014-05-15

**Authors:** Lina Kaminski, Jerry Eichler

**Affiliations:** Department of Life Sciences, Ben Gurion University, Beersheva, Israel; University of Alberta, Canada

## Abstract

In the halophilic archaea *Haloferax volcanii*, the surface (S)-layer glycoprotein can be modified by two distinct N-linked glycans. The tetrasaccharide attached to S-layer glycoprotein Asn-498 comprises a sulfated hexose, two hexoses and a rhamnose. While Agl11-14 have been implicated in the appearance of the terminal rhamnose subunit, the precise roles of these proteins have yet to be defined. Accordingly, a series of *in vitro* assays conducted with purified Agl11-Agl14 showed these proteins to catalyze the stepwise conversion of glucose-1-phosphate to dTDP-rhamnose, the final sugar of the tetrasaccharide glycan. Specifically, Agl11 is a glucose-1-phosphate thymidylyltransferase, Agl12 is a dTDP-glucose-4,6-dehydratase and Agl13 is a dTDP-4-dehydro-6-deoxy-glucose-3,5-epimerase, while Agl14 is a dTDP-4-dehydrorhamnose reductase. Archaea thus synthesize nucleotide-activated rhamnose by a pathway similar to that employed by Bacteria and distinct from that used by Eukarya and viruses. Moreover, a bioinformatics screen identified homologues of *agl11-14* clustered in other archaeal genomes, often as part of an extended gene cluster also containing *aglB*, encoding the archaeal oligosaccharyltransferase. This points to rhamnose as being a component of N-linked glycans in Archaea other than *Hfx. volcanii*.

## Introduction

N-glycosylation, the covalent attachment of oligosaccharides to select asparagine residues, is performed by members of all three domains of life [1]–[5]. Still, understanding of the archaeal version of this protein-processing event remains relatively limited. In the last decade, however, substantial progress has been realized in deciphering pathways of N-glycosylation in several archaeal species, including the halophile *Haloferax volcanii* [5].

In *Hfx. volcanii*, the surface (S)-layer glycoprotein, a well-studied glycoprotein and the sole component of the protein-based shell surrounding the cell, is modified by a pentasaccharide comprising a hexose, two hexuronic acids, a methyl ester of hexuronic acid and mannose. Through a series of genetic and biochemical studies, a series of Agl (*a*rchaeal *gl*ycosylation) proteins involved in the assembly and the attachment of this glycan to the S-layer glycoprotein Asn-13 and Asn-83 positions was described [6]–[12]. Most recently, a second glycan composed of a sulfated hexose, two hexoses and a rhamnose was shown to be N-linked to position Asn-498 of the S-layer glycoprotein [13]. Moreover, whereas the Asn-13- and Asn-83-linked pentasaccharide was identified when cells were grown across a range of NaCl concentrations, the novel Asn-498-bound tetrasaccharide was observed when cells were grown in 1.75 M but not 3.4 M NaCl-containing medium.

Relying on bioinformatics, gene deletions and mass spectrometry, Agl5-Agl15 have been identified as components of the pathway responsible for the assembly of the so-called ‘low salt’ tetrasaccharide N-linked to S-layer glycoprotein Asn-498 [14]. Based on these studies, Agl11-Agl14 were deemed to be involved in the appearance of the final sugar of the ‘low salt’ tetrasaccharide, rhamnose, on the dolichol-phosphate carrier upon which the glycan is initially assembled.

**Figure 1:**
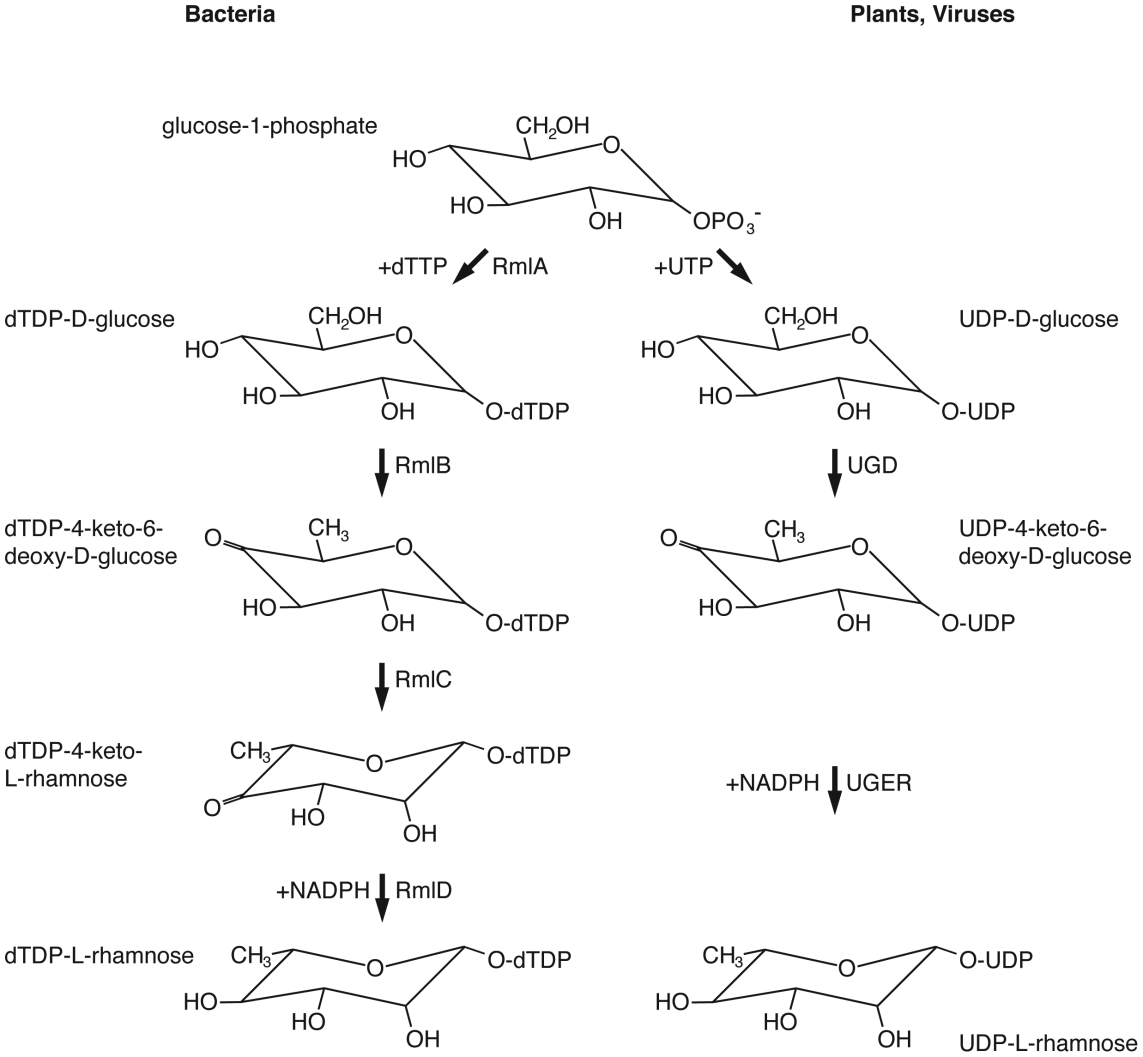
Pathways of nucleotide-activated rhamnose biogenesis. Schematic depiction of pathways of dTDP-rhamnose biosynthesis in Bacteria and of UDP-rhamnose biosynthesis in plants and viruses. UGD, UDP-D-glucose-4,6-dehydratase; UGER, UDP-4-keto-6-deoxy-D-glucose-3,5-epimerase/4-reductase.

Rhamnose, a naturally occurring deoxy-hexose, is found in the L- rather than the D-configuration assumed by most other sugars. In Bacteria, plants and fungi, rhamnose is a common component of the cell wall [15]–[17], and was also recently found in viruses [18]. At present, two pathways for synthesizing nucleotide-activated rhamnose are known. In Bacteria, RmlA, RmlB, RmlC and RmlD act sequentially to convert glucose-1-phosphate and deoxy-thymidine triphosphate (dTTP) into thymidine diphosphate (dTDP)-rhamnose [19], [20]. Specifically, RmlA, the first enzyme of the pathway, is a glucose-1-phosphate thymidylyltransferase that combines thymidine monophosphate with glucose-1-phosphate to create dTDP-glucose. RmlB, a dTDP-glucose-4,6-dehydratase, then catalyzes the oxidation and dehydration of dTDP-glucose to form dTDP-4-keto 6-deoxy-glucose. RmlC, a dTDP-4-dehydro-6-deoxy-glucose-3,5-epimerase, next performs a double epimerization at the C3 and C5 positions of the sugar. Finally, RmlD, a dTDP-4-dehydrorhamnose reductase, catalyzes the last step of the pathway, namely reduction of the C4 keto group of the sugar to yield dTDP-rhamnose. In plants, uridine diphosphate (UDP)-rhamnose rather than dTDP-rhamnose is generated by RHM (UDP-L-rhamnose synthase), a single polypeptide that contains all of the enzymatic activities required [21]. Here, UDP-glucose is converted to UDP-4-keto-6-deoxy-glucose by an enzymatic activity similar to bacterial RmlB. Next, and in contrast to the bacterial process, whereby RmlC and RmlD operate sequentially to generate dTDP-rhamnose, plants instead rely on nucleotide-rhamnose synthase/epimerase-reductase, a bifunctional enzyme mediating both the epimerization and reduction reactions that lead to the biosynthesis of UDP-rhamnose [21]–[23]. More recently, the same pathway was shown to catalyze UDP-rhamnose biogenesis in large DNA viruses [18]. The two pathways for nucleotide-activated rhamnose biosynthesis are depicted in Fig 1.

Although rhamnose has been identified in several archaeal species [13], [24], [25], studies addressing rhamnose biosynthesis in Archaea are few. In *Sulfolobus tokodaii*, one of three RmlA homologues was shown to possess sugar-1-phosphate nucleotydyltransferase activity using either glucose-1-phosphate or N-acetylglucosamine-1-phosphate and all four deoxyribonucleoside triphosphates or UTP as substrates [26], while *S. tokodaii* RmlB and RmlD were reported to be functionally identical to their bacterial counterparts [27]. At the same time, the crystal structure of *Methanobacter thermautotrophicus* RmlC has been reported [28], as have those of *S. tokodaii* RmlC and RmlD (PDB 2B9U and 2GGS, respectively). Still, it remains to be determined whether rhamnose is used for glycosylation by these species. Thus, to better understand the biosynthesis of this deoxy-hexose in Archaea, the present study addressed the involvement of *Hfx. volcanii* Agl11-Agl14 in the biogenesis of nucleotide-activated rhamnose. In addition, the presence and genomic distribution of homologues of genes involved in such activity across the Archaea were considered.

## Methods and Materials

### Chemicals

DNaseI, Glucose-1-phosphate, dTTP, dTDP-D-glucose, malachite green reagent, NADPH, phenylmethanesulfonyl fluoride (PMSF), pyrophosphatase, UTP and UDP-glucose were obtained from Sigma-Aldrich (St. Louis MO), dTDP-4-keto-6-deoxy-glucose came from Carbosynth (Berkshire, UK), novobiocin and ampicillin were obtained from Duchefa Biochemie (Haarlem, The Netherlands), while restriction endonucleases were purchased from Promega (Madison, WI).

### Strains and growth conditions

*Hfx. volcanii* WR536 (H53) parent strain cells were grown in complete medium containing 1.75 M NaCl, 0.15 M MgSO_4_•7H_2_0, 1 mM MnCl_2_, 4 mM KCl, 3 mM CaCl_2_, 0.3% (w/v) yeast extract, 0.5% (w/v) tryptone, 50 mM Tris-HCl, pH 7.2, at 42°C [14]. *Escherichia coli* were grown in Luria-Bertani medium at 37°C. Strains transformed to express plasmid-encoded versions of Agl11-Agl14 containing an N-terminally fused *Clostridium thermocellum* cellulose-binding domain (CBD) were supplemented with 100 µg/ml of ampicilin (for *E. coli*) or 1 µg/ml of novobiocin (for *Hfx. volcanii*).

### Plasmid construction

To generate a plasmid encoding CBD-Agl11, the *agl11* gene was PCR-amplified using primers designed to introduce NdeI and KpnI restriction sites at the 5′ and 3′ ends of the gene, respectively (primers listed in Table 1). The amplified fragment was digested with NdeI and KpnI and ligated into plasmid pWL-CBD, previously digested with the same restriction enzymes, to produce plasmid pWL-CBD-Agl11. Plasmid pWL-CBD-Agl11 was then introduced into *Hfx. volcanii* cells. Plasmids encoding CBD-Agl12, CBD-Agl13 and CBD-Agl14 were similarly generated, using the primers listed in Table 1, and also introduced into *Hfx. volcanii* parent strain cells.

**Table 1:** Primers used in this study.

Primer	Sequence^1^
*agl11*-NdeI-F	gggcatATGAAAGGCGTACTTCTCTCAGGAGG
*agl11*-KpnI-R	cccggtaccTTAGAGTTTCAGTTGGGAGTTCTC
*agl12*-NdeI-F	gggcatATGGACGTACTCGTTACTGGTGGTG
*agl12*-KpnI-R	cccggtaccCTATTCGTCGTCACCGAGGTAG
*agl13*-NdeI-F	gggcatATGCCAAACATCCACGATGTCG
*agl13*-KpnI-R	cccggtaccTTAGCCGTGGATTTCCGCGTTC
*agl14*-NdeI-F	gggcatATGTACGCATTCGTCACCGGC
*agl14*-KpnI-R	cccggtaccCTACGAGCTGTAATCGCTGAACG
*agl13F*	ATGCCAAACATCCACGATGTCG
*agl11R*	TTAGAGTTTCAGTTGGGAGTTCTCG

1 Genomic sequence in capital letters.

### Protein purification

To purify the CBD-tagged proteins, 1 ml aliquots of *Hfx. volcanii* cells transformed to express CBD-Agl11, CBD-Agl12, CBD-Agl13 or CBD-Agl14 were grown to mid-logarithmic phase, harvested and resuspended in 1 ml solubilization buffer (1% Triton X-100, 1.75 M NaCl, 50 mM Tris-HCl, pH 7.2) containing 3 µg/ml DNaseI and 0.5 µg/ml PMSF. The solubilized mixture was nutated for 20 min at 4°C, after which time 50 µl of a 10% (w/v) solution of cellulose was added. After a 120 min nutation at 4°C, the suspension was centrifuged (5,000 rpm for 5 min), the supernatant was discarded and the cellulose pellet was washed four times with wash buffer containing 1.75 M NaCl, 50 mM Tris-HCl, pH 7.2. After the final wash, the cellulose beads were centrifuged (5,000 rpm for 5 min), the supernatant was removed and the pellet, containing cellulose beads linked to CBD-tagged Agl11, Agl12, Agl13 or Agl14, was either subjected to further in vitro assays or resuspended in SDS-PAGE sample buffer, boiled for 5 min, centrifuged (5,000 rpm for 5 min) and subjected to SDS-PAGE and Coomassie Brilliant Blue staining.

### Agl11 activity assay

Cellulose-bound CBD-Agl11 were resuspended in reaction buffer containing 1.75 M NaCl, 5 mM MgCl_2_, 50 mM Tris-HCl, pH 7.2 and incubated with 5 mM glucose-1-phosphate and 5 mM dTTP (or UTP) at 42°C. As controls, glucose-1-phosphate, dTTP (or UTP) or both were omitted from the reaction. Aliquots were removed immediately following substrate addition and at several time points up to 40 min and incubated for 10 min at room temperature (RT) with 1 U/µl of pyrophosphatase. The extent of phosphate release was determined using a malachite green-based assay [29]. Briefly, 10 µl aliquots were incubated for 5 min at RT with 850 µl of a Malachite green solution followed by addition of 100 µl of 34% citric acid and incubation for an additional 40 min at RT. Phosphate concentration was calculated using a standard curve based on the 660 nm absorbance of a 0-1000 µM phosphate solution.

### Thin layer chromatography

To perform TLC, 10 µl of the products generated in the Agl11 assay described above were spotted onto a Partisil K6 silica gel plate (Whatman, Maidstone, UK). In addition, 10 µl of 2 mM glucose-1-phosphate and dTDP-D-glucose solutions were applied to the same plate as standards. The plates were developed in 95% ethanol/1 M acetic acid (5∶2, pH 7.5). The separated spots were detected by spraying the plate with orcinol monohydrate solution (0.1% in 5% H_2_SO_4_ in ethanol) and then heating the plate for 10 min at 120°C.

### Agl12 activity assay

The dTDP-D-glucose 4,6-dehydratase of Agl12 activity was assayed as described previously [30]. Briefly, cellulose-bound CBD-Agl12 was resuspended in reaction buffer containing 1.75 M NaCl, 5 mM MgCl_2_, 50 mM Tris-HCl, pH 7.2 and incubated at 42°C with 4 mM dTDP-D-glucose or UDP-D-glucose. Aliquots were removed immediately following substrate addition and at several time points up to 40 min and mixed with 750 µl of 100 mM NaOH. Each mixture was incubated for 20 min at 42°C and absorbance at 320 nm was measured.

### Combined Agl13 and Agl14 activity assay

Cellulose-bound CBD-Agl13 and CBD-Agl14 were resuspended in reaction buffer containing 1.75 M NaCl, 50 mM Tris-HCl, pH 7.2 and incubated with 4 mM dTDP-4-keto-6-deoxy-glucose and 10 mM NADPH at 42°C for 20 h. As controls, CBD-Agl13, CBD-Agl14 or NADPH was omitted. After incubation, the mixtures were centrifuged (5,000 rpm for 5 min), and the supernatant was examined by nano- ESI/MS analysis. For nano-ESI/MS analysis, a 10 µl aliquot was dried using a SpeedVac apparatus, resuspended in 10 µl methanol:water (1∶1; v/v) containing 10 mM ammonium acetate and injected into a LTQ Orbitrap XL mass spectrometer using static medium NanoES Spray capillaries (Thermo Fisher Scientific, Bremen, Germany). Mass spectra were obtained in the negative mode.

### Reverse transcriptase polymerase chain reaction (RT-PCR)

RT-PCR performed as previously described [31]. Briefly, RNA from *Hfx. volcanii* cells was isolated using TRIzol reagent (Invitrogen, Carlsbad, CA). cDNA was prepared for each sequence from the corresponding RNA (2 µg) using random hexamers (150 ng) in a SuperScript III First-Strand Synthesis System for RT-PCR (Invitrogen). The single-stranded cDNA was then used as PCR template in a reaction containing forward and reverse primers to sequences within *agl13* and *agl11*, respectively (Table 1). In control reactions, genomic DNA or RNA served as template, or no nucleic acid was added to the reaction. The generation of PCR products was assessed by electrophoresis in 1% agarose followed by detection using ethidium bromide.

### Bioinformatics analysis

Predicted archaeal RmlABCD proteins were identified using *Hfx. volcanii* Agl11, Agl12, Agl13 and Agl14 as query in a BLAST search of the Joint Genome Institute Database for Integrated Microbial Genomes – Expert Review (https://img.jgi.doe.gov/cgi-bin/er/main.cgi), using the terms ‘EC 2.7.7.24’, ‘EC 4.2.1.46’, ‘EC 5.1.3.13’ and ‘EC 1.1.1.133’ to search for RmlA, RmlB, RmlC and RmlD homologues, respectively. Archaeal RmlA-, RmlB-, RmlC- and RmlD-encoding genes were deemed as being clustered with the oligosaccharyltransferase-encoding *aglB* gene based upon the presence of these genes within previously identified *aglB*-based glycosylation gene clusters [32], or when *rmlA*, *rmlB*, *rmlC* and *rmlD* were clustered and found 10 genes or less away from clusters containing *aglB* and other glycosylation- or sugar processing-related genes.

## Results

### Agl11 is a glucose-1-phosphate thymidylyltransferase/uridylyltransferase

**Figure 2:**
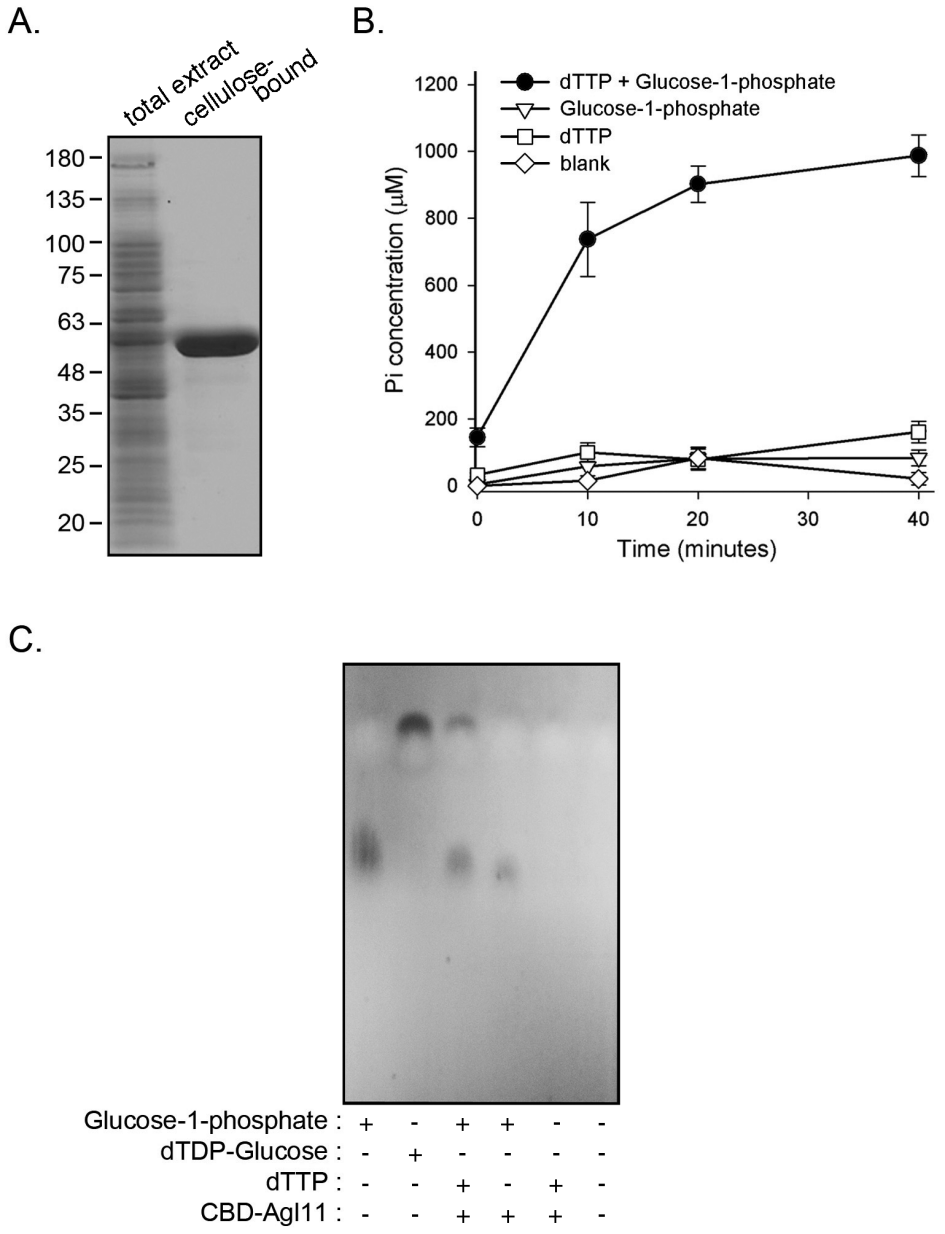
Agl11 is a glucose-1-phosphate thymidylyltransferase. A. *Hfx. volcanii* cells transformed to express CBD-Agl11 were subjected to cellulose-based chromatography. A cell extract and cellulose-bound proteins were separated on 10% SDS-PAGE and Coomassie-stained. A cellulose-bound ∼55 kDa protein band corresponding to CBD-Agl11 is observed. The positions of molecular weight markers are shown on the left. B. Cellulose-bound CBD-Agl11 or cellulose beads alone (blank) were resuspended in reaction buffer and incubated in the presence of dTTP and glucose-1-phosphate, with each substrate separately or without both substrates. Aliquots removed immediately after substrate addition and up to 40 min later were incubated with pyrophosphatase and the extent of phosphate release was measured 29]. The results represent average of triplicates ± standard deviation for one of three repeats of the experiment. C. The assay products obtained after a 5 h incubation at 42°C were separated by TLC, along with glucose-1-phosphate and dTDP-glucose standards, as described in Experimental Procedures.

As a first step towards defining the precise function of Agl11, a BLAST homology-based search was conducted using *Hfx. volcanii* Agl11 as query. This revealed the homology of Agl11 to RmlA, the bacterial glucose-1-phosphate thymidylyltransferase (EC 2.7.7.24) that catalyzes the formation of dTDP-glucose from dTTP and glucose 1-phosphate [33]. For instance, Agl11 shared 53% identity, with 100% coverage and an E-value of 8e-120, to RmlA from the bacterium *Sulfobacillus acidophilus* TPY. To biochemically confirm that Agl11 indeed acts as does RmlA, *Hfx. volcanii* cells were transformed with a plasmid encoding Agl11 bearing an N-terminally-fused CBD tag [34]. The presence of the CBD tag allows for cellulose-based purification compatible with the hypersaline conditions in which *Hfx. volcanii* grow. PCR amplification using DNA extracted from the transformed strain as template, together with forward and reverse primers directed against regions within the *CBD* and *agl11* sequences, respectively, confirmed uptake of the plasmid (not shown). Cellulose-based purification of an extract prepared from the transformed cells captured a single 55 kDa protein, corresponding to the predicted molecular mass of the 17 kDa CBD moiety and the 38 kDa Agl11 protein (Fig 2A).

The predicted glucose-1-phosphate thymidylyltransferase activity of purified Agl11 was next considered. Glucose-1-phosphate thymidylyltransferase, like RmlA, transfers the deoxy-thymidine monophosphate (dTMP) group of dTTP to glucose-1-phosphate to yield dTDP-glucose and pyrophosphate. Hence, the actions of Agl11 as a glucose-1-phosphate thymidylyltransferase was tested using a malachite green-based assay to detect the formation of phosphate following the conversion of pyrophosphate into inorganic phosphate upon addition of pyrophosphatase [29]. The assay revealed that Agl11 was able to generate phosphate only when incubated with dTTP and glucose-1-phosphate but not with either substrate alone or without both substates (Fig 2B). Thin layer chromatography (TLC) was also employed to further confirm the glucose-1-phosphate thymidylyltransferase activity of Agl11. In these experiments, the product generated upon incubation of Agl11 with dTTP and glucose-1-phosphate migrated to the same position as a dTDP-glucose standard (Fig 2C). Similar results were obtained when UTP was used in place of dTTP (not shown). As such, Agl11 acts as a glucose-1-phosphate thymidylyltransferase and a glucose-1-phosphate uridylyltransferase, namely the first enzyme in the biosynthesis of nucleotide activated-rhamnose in bacteria and in plants, respectively.

### Agl12 is a dTDP-glucose-4,6-dehydratase

The function of Agl12 was next addressed. A BLAST homology-based search revealed the homology of *Hfx. volcanii* Agl12 to both dTDP-glucose-4,6-dehydratase (RmlB) (EC 4.2.1.46), the bacterial enzyme catalyzing the second step of the dTDP-rhamnose biosynthetic pathway, i.e, the conversion of dTDP-glucose to dTDP-4-keto-6-deoxy-glucose [35], and to UDP-glucose-4-epimerase (or UDP-galactose-4-epimerase; EC 5.1.3.2), the enzyme catalyzing the reversible conversion of UDP-galactose to UDP-glucose, the final step in the Leloir pathway of galactose metabolism [36]. Specifically, Agl12 shared 58% identity, with 98% coverage and an E-value of 5e-125, to RmlB from the bacterium *Caldithrix abyssi.* Indeed, Agl12 contains the GxxGxxG (^7^GGAGFIG^13^) and YxxxK (^146^YSATK^150^) motifs characteristic of RmlB proteins [23].

**Figure 3:**
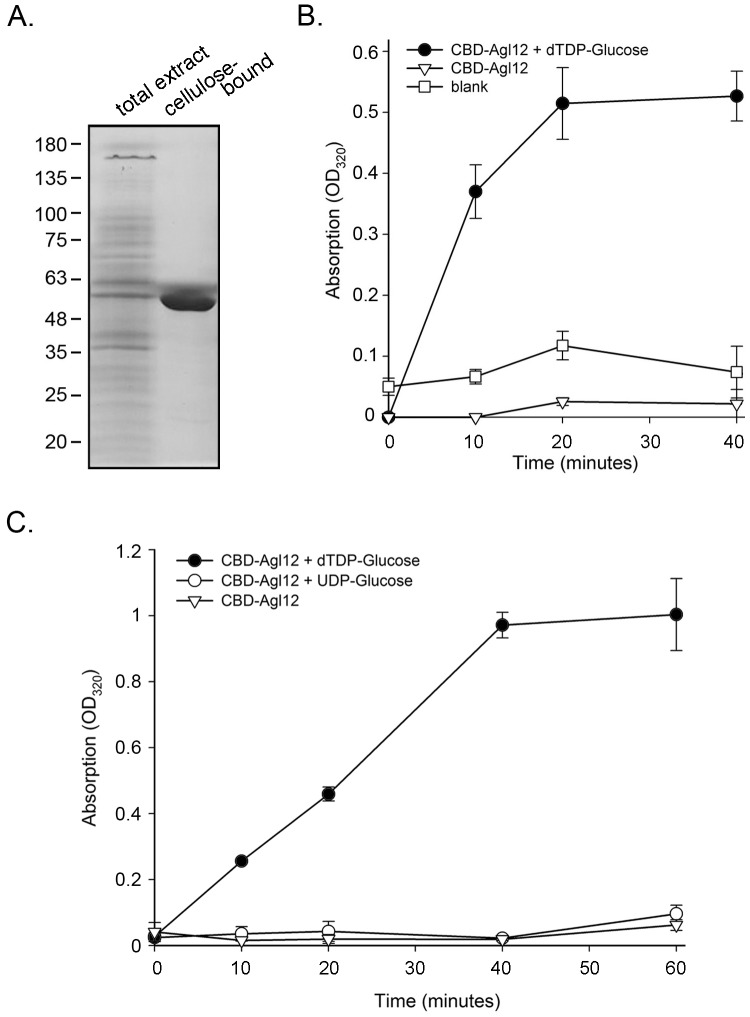
Agl12 functions as dTDP-D-glucose-4,6-dehydratase. A. *Hfx. volcanii* cells transformed to express CBD-Agl12 were subjected to cellulose-based capture. A cell extract and cellulose-bound protein were separated on 10% SDS-PAGE and Coomassie-stained. Protein band corresponding to CBD-Agl11 (∼51 kDa) is observed. The positions of molecular weight markers are shown on the left of the gel. B. Cellulose-bound CBD-Agl12 or cellulose beads alone (blank) was resuspended in reaction buffer and incubated in the presence of dTDP-glucose or no substrate. Immediately after substrate addition and up to 40 min of incubation at 42°C, the samples were incubated with 0.1 M NaOH for 20 min at 42°C and the increase of absorbance at 320 nm was measured. C. The same assay was repeated for up to 60 min using dTDP-glucose or UDP-glucose as substrate or with no substrate. The assay results presented in B and C represent averages of triplicates ± standard deviation obtained in one of two repeats of the experiment.

To test whether Agl12 indeed acts as a dTDP-glucose-4,6-dehydratase, working downstream to Agl11 in the biosynthesis of dTDP-rhamnose, *Hfx. volcanii* cells were transformed to express CBD-tagged Agl12. Again, successful transformation was verified by PCR amplification using DNA from the transformed strain as template, together with forward and reverse primers directed against regions within the *CBD* and *agl12* sequences, respectively (not shown). Cellulose-based purification of an extract prepared from *Hfx. volcanii* cells transformed to express CBD-Agl12 captured a 51 kDa species, corresponding to the predicted molecular mass of the 17 kDa CBD moiety and the 34 kDa Agl12 protein (Fig 3A).

Cellulose-purified CBD-Agl12 was incubated in the absence or presence of dTDP-glucose, the product of the Agl11-catalyzed reaction, and the formation of dTDP-4-keto-6-deoxy-glucose was assessed spectrophotometrically, following the increase in absorption at 320 nm, indicative of the formation of the product keto group. dTDP-4-keto-6-deoxy-glucose was only generated when Agl12 was combined with dTDP-glucose, confirming that Agl12 is indeed a dTDP-glucose-4,6-dehydratase, like RmlB (Fig 3B). When CBD-Agl12 was combined with UDP-glucose, the product generated when UDP-rhamnose serves as substrate, no UDP-4-keto-6-deoxy-glucose was formed (Fig 3C).

### Agl13 and Agl14 are homologues of RmlC and RmlD, respectively

Next, the functions of Agl13 and Agl14 were addressed. The homologies of *Hfx. volcanii* Agl13 to dTDP-4-dehydrorhamnose-3,5-epimerase (RmlC) (EC 5.1.3.13) and of Agl14 to dTDP-4-dehydrorhamnose reductase (RmlD) (EC 1.1.1.133), bacterial enzymes respectively catalyzing the isomerization of dTDP-4-keto-6-deoxy-glucose to dTDP-4-keto-L-rhamnose and its dehydrogenation to yield dTDP-rhamnose [36], were revealed by BLAST searches. Specifically, Agl13 shared 51% identity, with 97% coverage and an E-value of 9e-45, to RmlC from *Nitrolancea hollandica*, while Agl14 shared 37% identity, with 96% coverage and an E-value of 1e-46, to RmlD from *Ammonifex degensii* KC4. Moreover, while Agl14 contains the GxxGxxG (^7^GANGLLG^13^) and YxxxK (^135^YGRSK^139^) motifs characteristic of RmlD proteins [37].

**Figure 4:**
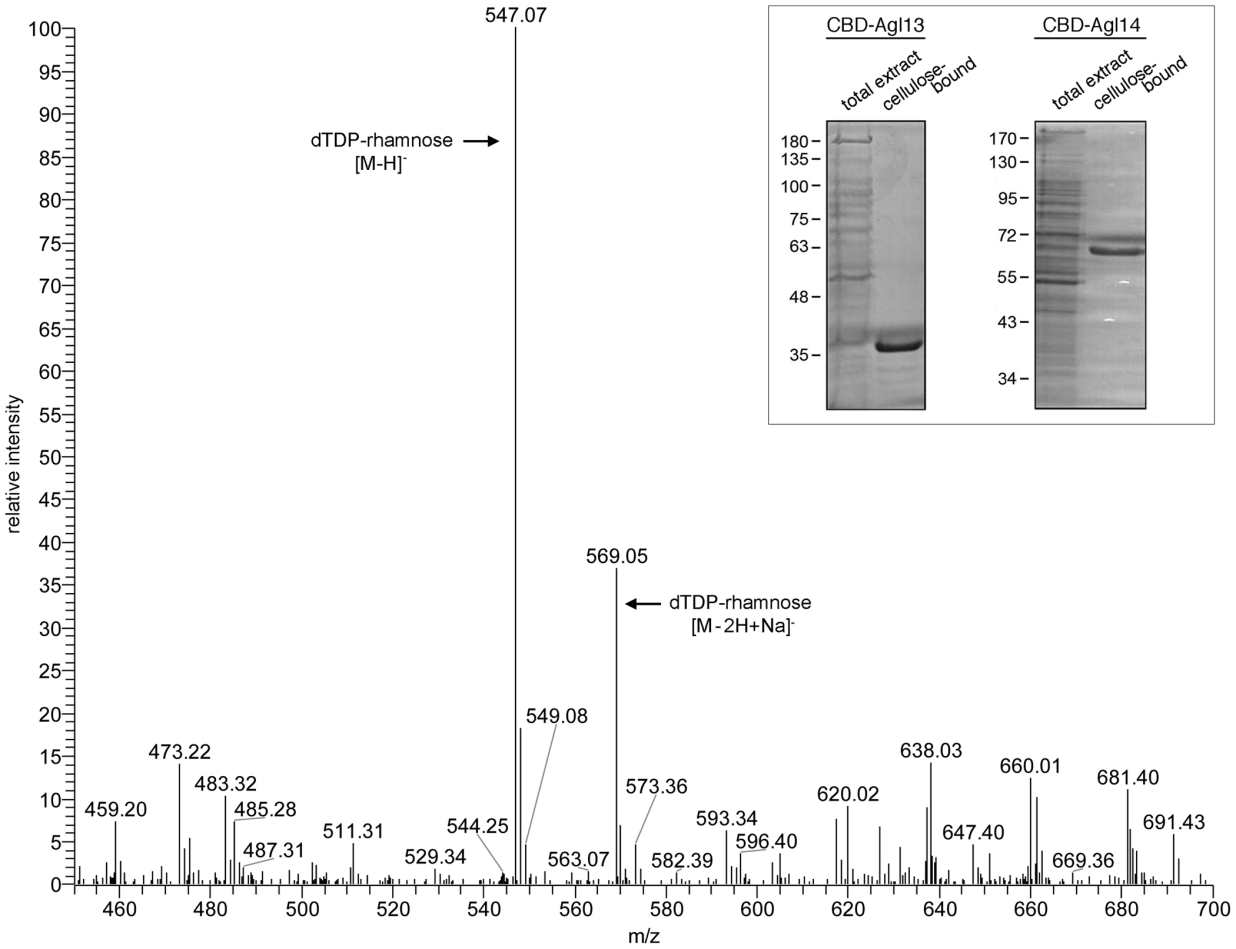
Agl13 and Agl14 together convert dTDP-4-keto-6-deoxy-glucose into dTDP-rhamnose. Cellulose-bound CBD-Agl13 and CBD-Agl14 were combined with dTDP-4-keto-6-deoxy-glucose and NADPH and the soluble fraction was examined by nano- ESI/MS analysis. Peaks corresponding to dTDP-rhamnose and the sodium adduct are indicated. Inset: Purification of CBD-Agl13 and CBD-Agl14. Cell extracts and cellulose-bound protein from *Hfx. volcanii* cells transformed to express CBD-Agl13 (left) or CBD-Agl14 (right) were separated on 10% SDS-PAGE and Coomassie-stained. Protein bands corresponding to CBD-Agl13 and CBD-Agl14 are observed in each lane of cellulose-bound material. The positions of molecular weight markers are shown on the left of each gel.

To determine whether Agl13 and Agl14 indeed participate in the biosynthesis of dTDP-rhamnose by acting as RmlC and RmlD, respectively, *Hfx. volcanii* cells were transformed to express CBD-tagged versions of Agl13 and Agl14. Here as well, each transformation was verified by PCR amplification using DNA from the transformed strain as template, together with forward and reverse primers directed against regions within the *CBD* and *agl13*, or the *CBD* and *agl14* sequences, respectively (not shown). Following transformation, cellulose-based purification of extracts prepared from *Hfx. volcanii* cells transformed to express either CBD-Agl13 or CBD-Agl14 was conducted. Following SDS-PAGE separation of cellulose-captured proteins, only bands corresponding to CBD-Agl13 or CBD-Agl14 were observed (Fig 4 inset, left and right panels, respectively).

The ability of Agl13 and Agl14 to act as RmlC and RmlD respectively in the production of dTDP-rhamnose was next considered in a combined assay. Briefly, dTDP-4-keto-6-deoxy-glucose was incubated together with CBD-tagged Agl13 and Agl14, along with NADPH, the substrate for the dehydrogenase reaction putatively catalyzed by Agl14. The appearance of dTDP-rhamnose was revealed by nano-electrospray ionization mass spectrometry (nano-ESI/MS) [18], since initial attempts to detect dTDP-rhamnose formation spectrophotometrically as previously described [38], [39] were unsuccessful. Nano-ESI/MS analysis revealed the formation of a *m/z* 547.07 peak corresponding to dTDP-rhamnose (*m/z* 547.07 calculated [M-H]^−^ mass) and a peak at *m/z* 569.05 corresponding to the sodium adduct (*m/z* 569.05 calculated [M-2H+Na]^−^ mass) (Fig 4). In the absence of CBD-Agl13, CBD-Agl14 or NADPH, peaks corresponding to dTDP-4-keto-6-deoxy-glucose were observed (*m/z* 545.06 calculated [M-H]^−^ mass); no peaks corresponding to dTDP-rhamnose were seen (Fig S1A-C, respectively). In the absence of dTDP-4-keto-6-deoxy-glucose, no peaks corresponding to either sugar were detected (Fig S1D).

### agl11 and agl13 are co-transcribed

**Figure 5:**
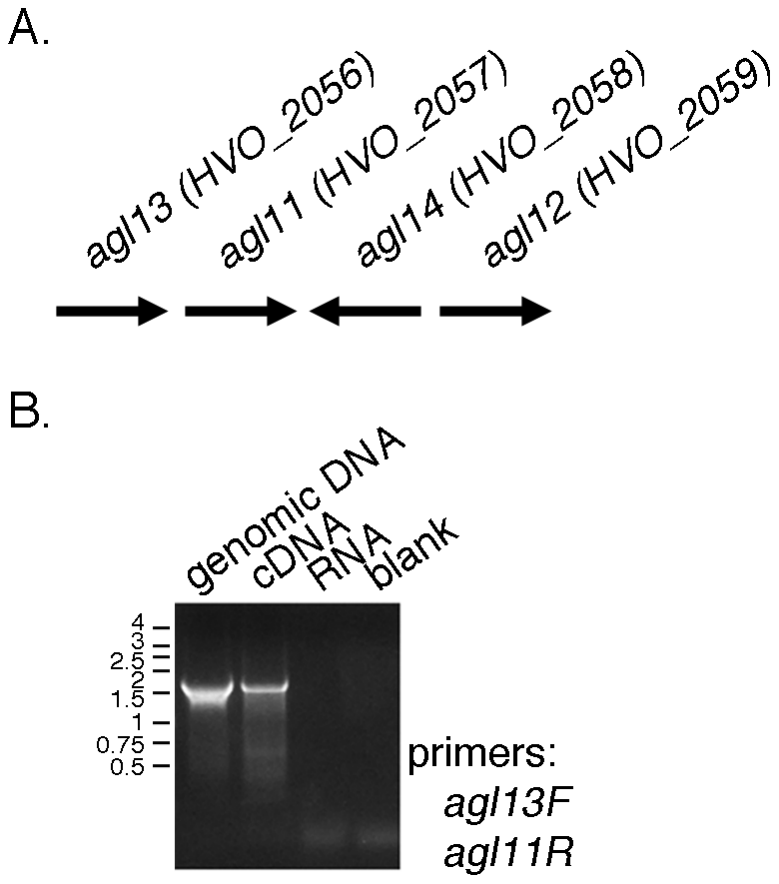
*agl11* and *agl13* are co-transcribed. A. Schematic depiction of the position and orientation of *agl11*-*agl14* in the *Hfx. volcanii* genome. The length of each gene is arbitrarily drawn. B. PCR amplifications were performed using a forward primer against a region within *agl11* and a reverse primer against a region within *agl13*, together with genomic DNA or RNA isolated from *Hfx. volcanii* cells, cDNA prepared from the same RNA or no nucleic acid (blank) as template. The positions of Kbp markers are shown on the left.

To obtain further insight into the actions of Agl11-Agl14, the transcription of each gene was addressed. Specifically, given that *agl11* is found adjacent to *agl13* in the *Hfx. volcanii* genome and that both are similarly oriented (Fig 5A), the co-transcription of these genes was considered. Accordingly, RT-PCR amplification was performed using primers directed at regions corresponding to the beginning of *agl13* and the end of *agl11* together with cDNA produced from RNA isolated from *Hfx. volcanii* cells. A PCR product of approximately 1500 bp, consistent with the genomic sizes of *agl13* (471 bp) and *agl11* (1074 bp), was observed (Fig 5B).

### Genome scanning reveals the clustering of rmlABCD in other Archaea

Given the identification of an *rmlABCD* gene cluster in *Hfx. volcanii*, similar clusters were sought in other available archaeal genomes. Towards this aim, the 166 completed archaeal genomes listed at the Joint Genome Institute Database for Integrated Microbial Genomes (January, 2014) were subjected to a BLAST search seeking homologues of *Hfx. volcanii* Agl11, Agl12, Agl13 and Agl14. In addition, these genomes were also scanned for genes encoding proteins listed as EC 2.7.7.24 (RmlA), EC 4.2.1.46 (RmlB), EC 5.1.3.13 (RmlC) or EC 1.1.1.133 (RmlD). In this manner, 69 genomes were shown to encode an *rmlABCD* gene cluster, including *Hfx. volcanii*. Of these, 16 included *rmlABCD* as part of a previously defined larger cluster anchored by *aglB*, encoding the archaeal oligosaccharyltransferase [32] (Table 2). In addition, 19 species were found to encode partial *rmlABCD* gene clusters, where two or three of these genes are clustered (Table S1). Of these species, four (*Methanobrevibacter ruminantium*, *Methanosarcina acetivorans*, *Sulfolobus islandicus* Y.G.57.14 and *Sulfolobus solfataricus* P2) also encode a complete *rmlABCD* gene cluster.

**Table 2:** Archaea where *rmlABCD* are clustered.

Species	*rmlA*	*rmlB*	*rmlC*	*rmlD*	*aglB* 1
*Acidilobus saccharovorans*	ASAC_0660	ASAC_0661	ASAC_0658	ASAC_0659	
*Aciduliprofundum boonei*	Aboo_0257	Aboo_0256	Aboo_0255	Aboo_0254	
*Aeropyrum pernix*	APE1181	APE1180	APE1178	APE1179	
*Archaeoglobus fulgidus* 7324	AFULGI_00003220	AFULGI_00003210	AFULGI_00003190	AFULGI_00003200	AFULGI_00003280
*Archaeoglobus fulgidus* VC-16	AF00325	AF00324	AF00323a	AF00323b	AF00329
*Archaeoglobus profundus*	Arcpr_1197	Arcpr_1198	Arcpr_1202	Arcpr_1201	Arcpr_1194
*Archaeoglobus veneficus*	Arcve_0544	Arcve_0545	Arcve_0551	Arcve_0546	Arcve_0568
*Caldisphaera lagunensis*	Calag_0942	Calag_0943	Calag_0941	Calag_0944	
*Candidatus Caldiarchaeum subterraneum*	Kcr_0845	Kcr_0846	Kcr_0848	Kcr_0847	
*Candidatus Methanomethylophilus alvus*	MMALV_00940	MMALV_00960	MMALV_00950	MMALV_00970	
*Candidatus Methanoregula boonei*	Mboo_1752	Mboo_1749	Mboo_1751	Mboo_1750	
*Candidatus Nitrosopumilus* sp. AR2	NSED_08565,08570	NSED_08580	NSED_08585	NSED_08575	
*Desulfurococcus mucosus*	Desmu_1148	Desmu_1145	Desmu_1143	Desmu_1144	
*Haloferax volcanii*	Agl11	Agl12	Agl13	Agl14	
*Halogeometricum borinquense*	Hbor_31500	Hbor_31470	Hbor_31510	Hbor_31480	
*Halomicrobium mukohataei*	Hmuk_1214	Hmuk_1217	Hmuk_1213	Hmuk_1216	
*Halophilic archaeon* sp. DL31	Halar_0604	Halar_0607	Halar_0603	Halar_0606	
*Halorhabdus utahensis*	Huta_2143	Huta_2146	Huta_2142	Huta_2145	
*Metallosphaera cuprina*	Mcup_0924	Mcup_0925	Mcup_0922	Mcup_0923	
*Methanobacterium* sp. SWAN-1	MSWAN_0553	MSWAN_0550	MSWAN_0551	MSWAN_0552	
*Methanobrevibacter ruminantium*	mru_0108	mru_0110	mru_0109	mru_0107	
*Methanobrevibacter smithii* PS	Msm_1307	Msm_1309	Msm_1308	Msm_1304	
*Methanocella arvoryzae*	MRE50lv_2499	MRE50lv_2497	MRE50lv_2496	MRE50lv_2498	
*Methanocella conradii*	Mtc_0188,185	Mtc_0186,189	Mtc_0190	Mtc_0187	
*Methanocella paludicola*	MCPlv_2757,2760	MCPlv_2756,2759	MCPlv_2755	MCPlv_2758	MCPlv_2762
*Methanococcoides burtonii*	Mbur_2230	Mbur_2232	Mbur_2233	Mbur_2231	
*Methanococcus aeolicus*	Maeo_0379	Maeo_0380	Maeo_0383	Maeo_0381	
*Methanococcus maripaludis* C5	MmarC5_1315	MmarC5_1314	MmarC5_1316	MmarC5_1313	
*Methanococcus maripaludis* C6	MmarC6_0591	MmarC6_0590	MmarC6_0592	MmarC6_0589	
*Methanococcus maripaludis* X1	GYY_01860	GYY_01865	GYY_01855	GYY_01875	
*Methanoculleus marisnigri*	Memar_0188	Memar_0186	Memar_0185	Memar_0187	Memar_0175
*Methanolobus psychrophilus*	Mpsy_2402	Mpsy_2400	Mpsy_2399	Mpsy_2401	
*Methanosaeta concilii*	MCON_2594	MCON_2593	MCON_2590	MCON_2591	
*Methanosaeta harundinacea*	Mhar_1098	Mhar_1099	Mhar_1097	Mhar_1100	Mhar_1091
*Methanosaeta thermophila*	Mthe_0954	Mthe_0956	Mthe_0955	Mthe_0953	
*Methanosarcina acetivorans*	MA3777	MA3779	MA3780	MA3778	MA3752,3753,3754
*Methanosarcina barkeri*	Mbar_A0233	Mbar_A0231	Mbar_A0230	Mbar_A0232	Mbar_A0242,A024
*Methanosarcina mazei*	MM1169	MM1167	MM1166	MM1168	
*Methanosphaerula palustris*	Mpal_2406	Mpal_2404	Mpal_2403	Mpal_2405	
*Methanospirillum hungatei*	Mhun_3075	Mhun_3072	Mhun_3074	Mhun_3073	Mhun_3066
*Methanothermobacter marburgensis*	MTBMA_c03630	MTBMA_c03610	MTBMA_c03620	MTBMA_c03640	
*Methanothermobacter thermoautotrophicus*	MTH1791	MTH1789	MTH1790	MTH1792	
*Methanothermus fervidus*	Mfer_0286	Mfer_0280	Mfer_0281	Mfer_0285	
*Methanotorris formicicus*	Metfo_1922	Metfo_1923	Metfo_1926	Metfo_1925	
*Methanotorris igneus*	Metig_0176	Metig_0177	Metig_0179	Metig_0178	
*Picrophilus torridus*	PTO0307	PTO0310,0312	PTO0311	PTO0308	
*Pyrococcus abyssi*	PAB0784	PAB0785	PAB0787	PAB0789	PAB1586
*Pyrococcus horikoshii*	PH0417	PH0414	PH0413	PH0417	
*Pyrococcus* sp. ST04	Py04_0479	Py04_0480	Py04_0481	Py04_0482	Py04_0456
*Pyrococcus yayanosii*	PYCH_17780	PYCH_17770	PYCH_17740	PYCH_17690	PYCH_17920
*Pyrolobus fumarli*	Pyrfu_0681	Pyrfu_0680	Pyrfu_0677	Pyrfu_0679	
*Sulfolobus acidocaldarius* 98-3	Saci_1703	Saci_1704	Saci_1706	Saci_1705	
*Sulfolobus acidocaldarius* N8	SacN8_08270	SacN8_08275	SacN8_08285	SacN8_08280	
*Sulfolobus acidocaldarius* Ron12/l	SacRon12l_08280	SacRon12l_08285	SacRon12l_08295	SacRon12l_08290	
*Sulfolobus islandicus* LAL14/1	Sil_0867	Sil_0868	Sil_0865	Sil_0866	
*Sulfolobus islandicus* L.D.8.5	LD85_1121	LD85_1117	LD85_1123	LD85_1122	
*Sulfolobus islandicus* REY15A	SiRe_0841	SiRe_0840	SiRe_0843	SiRe_0842	
*Sulfolobus islandicus* Y.G.57.14	YG5714_0665	YG5714_0664	YG5714_0667	YG5714_0666	
*Sulfolobus solfataricus* P2	SSO0831	SSO0830	SSO0833	SSO0832	
*Thermococcus barophilus*	TERMP_02079	TERMP_02080	TERMP_02084	TERMP_02089	TERMP_02078
*Thermococcus onnurineus*	TON_1842	TON_1843	TON_1848	TON_1851	TON_1820
*Thermococcus sibiricus*	TSIB_2044	TSIB_2045	TSIB_2047	TSIB_2048	TSIB_0007
*Thermogladius cellulolyticus*	TCELL_0180	TCELL_0179	TCELL_0177	TCELL_0178	
*Thermogladius shockii*	Des1633_00001920	Des1633_00001910	Des1633_00001890	Des1633_00001900	
*Thermoproteus tenax*	TTX_1336	TTX_1335	TTX_1333	TTX_1334	
*Thermosphaera aggregans*	Tagg_0563	Tagg_0562	Tagg_0560	Tagg_0561	

1 Clustering with *aglB* is defined as occurring when *rmlABCD* are part of a gene cluster containing *aglB* as described in ref. [39] or ≤10 genes away from such *aglB*–based clusters.

## Discussion

In addition to the pentasaccharide linked to select Asn residues of the *Hfx. volcanii* S-layer glycoprotein, it was recently shown that at least one additional Asn can be modified by a novel tetrasaccharide [14]. While many of the enzymes involved in the assembly of the N-linked pentasaccharide have been characterized biochemically [9], [10], [12], [40], virtually nothing is known of the enzymes responsible for the assembly of the N-linked tetrasaccharide. As such, this study reports the first biochemical analysis of enzymes contributing to this novel N-glycosylation pathway. The results reveal that Agl11 is a glucose-1-phosphate thymidylyltransferase, Agl12 is a dTDP-glucose-4,6-dehydratase, Agl13 is a dTDP-4-dehydro-6-deoxy-glucose-3,5-epimerase and Agl14 is a dTDP-4-dehydrorhamnose reductase.

While rhamnose is a common component of both the bacterial and the plant cell wall, different biosynthetic pathways are employed in each case, leading to the generation of differentially nucleotide-activated species. At the same time, it is not clear which of these strategies Archaea employ for nucleotide-activated rhamnose biogenesis. Indeed, numerous examples of Archaea relying on the same biochemical pathways as used by either their bacterial or eukaryal counterparts have been reported, as have examples of archaeal pathways comprising selected aspects of the parallel bacterial and eukaryal processes or even biosynthetic pathways unique to this form of life [41]–[50]. In the case of *Hfx. volcanii*, the current study revealed that Agl11-Agl14 are homologous to RmlA-D, enzymes that catalyze the conversion of glucose-1-phosphate to dTDP-rhamnose in Bacteria [19], [20]. Indeed, examination of available archaeal genomes detected the presence of RmlA-D in numerous species, pointing to Archaea and Bacteria as relying on the same route for nucleotide-activated rhamnose generation. At the same time, no gene encoding a homologue of the bifunctional nucleotide-rhamnose synthase/epimerase-reductase used in eukaryal UDP-rhamnose biosynthesis was detected in Archaea. Still, the fact that several archaeal species encode only a partial *rmlABCD* cluster (Table S1) raises the possibility that those enzymes present are recruited for the synthesis of molecules other than dTDP-rhamnose.

In addition to determining the route of nucleotide-activated rhamnose biosynthesis in *Hfx. volcanii*, the present study also represents the first biochemical characterization of components of a second N-glycosylation pathway recently identified in this species [14]. Based on earlier work revealing the presence of one or more genes encoding AglB, the oligosaccharyltransferase of the archaeal N-glycosylation machinery, in all but two of 168 genomes considered, it would appear that this protein-processing event is common in Archaea [40]. Yet, the diverse composition of the few N-linked archaeal glycans characterized to date points to archaeal N-glycosylation as largely relying on species-specific pathways [5]. The finding that some species contain *rmlABCD* homologues as part of a larger gene cluster containing *aglB* and other sugar-related genes implies that as in *Hfx. volcanii*, rhamnose is a component of N-linked glycans in these other Archaea as well. Continued investigation into archaeal protein glycosylation will test this prediction.

Finally, the simultaneous modification of the same protein by two completely different N-linked glycans has only been reported to date in two haloarchaeal species, namely *Halobacterium salinarum* and *Hfx. volcanii* [13], [51]. Of these, it is only in *Hfx. volcanii* that two N-glycosylation pathways have been identified [14]. Moreover, it was shown that N-glycosylation by both pathways occurs as a function of salt levels in the growth medium [13]. At present, it is not clear why the *Hfx. volcanii* S-layer glycoprotein is modified by two distinct N-linked glycans in 1.75 M NaCl-containing medium but not when cells are grown at higher salinity, nor what advantages such differential N-glycosylation offer the cell. The results obtained in this study will help answer these and other outstanding questions related to *Hfx. volcanii* N-glycosylation.

## Supporting Information

Figure S1 **Agl13, Agl14 and NADPH are required for the conversion of dTDP-4-keto-6-deoxy-glucose into dTDP-rhamnose.** Reactions were conducted as described in the legend to Figure 4, albeit in the absence of cellulose-bound CBD-Agl13 (A), CBD-Agl14 (B) or NADPH (C). In each case, nano-ESI/MS analysis detected peaks corresponding to dTDP-4-keto-6-deoxy-glucose (*m/z* 545.06 calculated [M-H]^−^ mass) but not peaks corresponding to dTDP rhamnose (*m/z* 547.07 calculated [M-H]^−^ mass). When the reaction was conducted in the absence of dTDP-4-keto-6-deoxy-glucose (D), no peaks corresponding to either sugar were detected. As standards, 10 µl of 1 mM dTDP-4-keto-6-deoxy-glucose (E) and dTDP rhamnose (F) solutions were examined by nano-ESI/MS.
(TIF)

Table S1 **Archaea encoding partial** ***rmlABCD*** **clusters.**
(DOC)
