# Supplementary figures and images for: Haloferax volcanii N-Glycosylation: Delineating the Pathway of dTDP-rhamnose Biosynthesis
Source: PLoS One. 2014 May 15;9(5):e97441. doi: 10.1371/journal.pone.0097441 (PMC4022621; doi:10.1371/journal.pone.0097441)

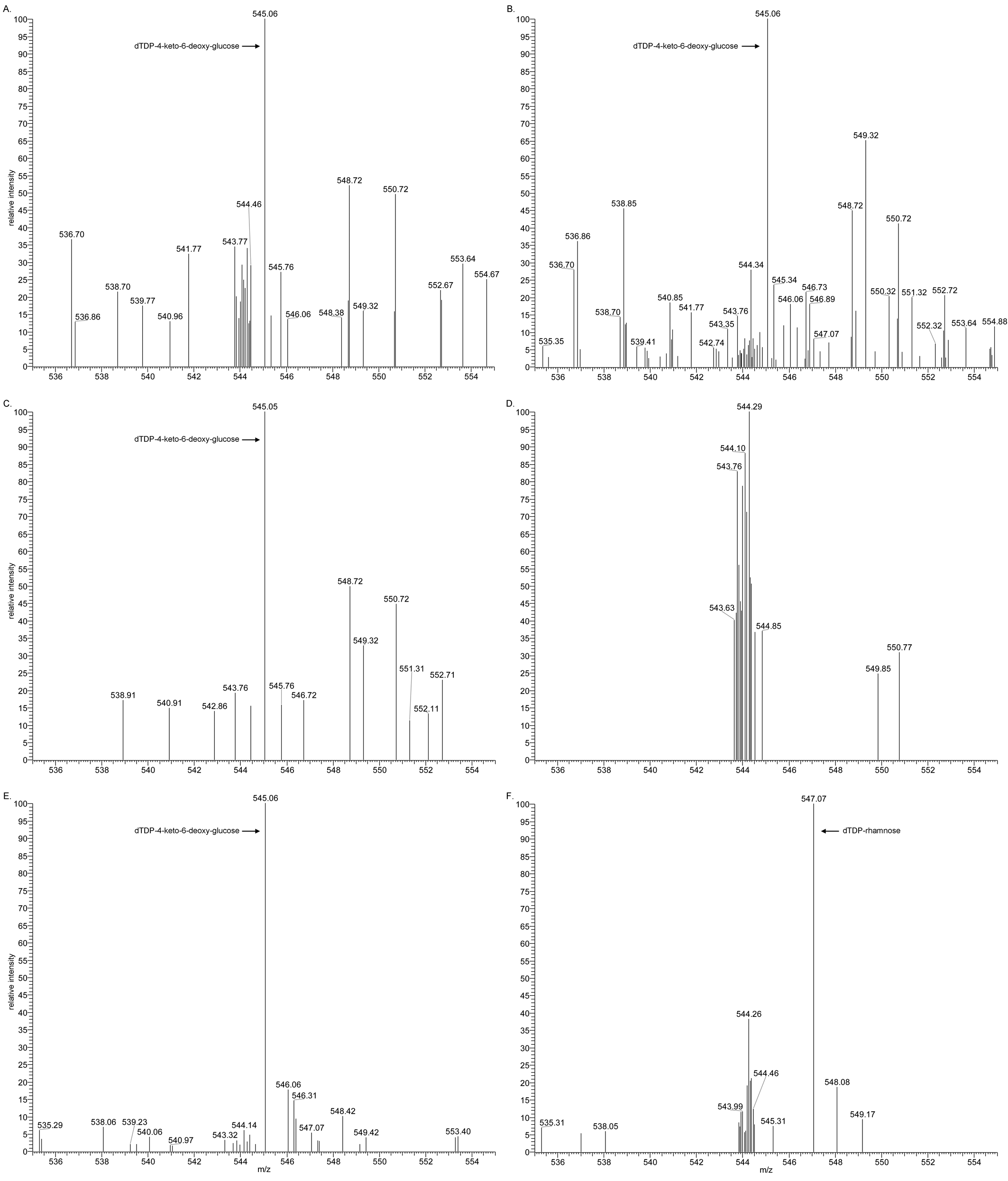

Supplement: Figure S1 — Agl13, Agl14 and NADPH are required for the conversion of dTDP-4-keto-6-deoxy-glucose into dTDP-rhamnose. Reactions were conducted as described in the legend to Figure 4, albeit in the absence of cellulose-bound CBD-Agl13 (A), CBD-Agl14 (B) or NADPH (C). In each case, nano-ESI/MS analysis detected peaks corresponding to dTDP-4-keto-6-deoxy-glucose (m/z 545.06 calculated [M-H]− mass) but not peaks corresponding to dTDP rhamnose (m/z 547.07 calculated [M-H]− mass). When the reaction was conducted in the absence of dTDP-4-keto-6-deoxy-glucose (D), no peaks corresponding to either sugar were detected. As standards, 10 µl of 1 mM dTDP-4-keto-6-deoxy-glucose (E) and dTDP rhamnose (F) solutions were examined by nano-ESI/MS. (TIF) [file pone.0097441.s001.tif]
