# Supplementary material for: Haloferax volcanii N-Glycosylation: Delineating the Pathway of dTDP-rhamnose Biosynthesis
Source: PLoS One. 2014 May 15;9(5):e97441. doi: 10.1371/journal.pone.0097441 (PMC4022621; doi:10.1371/journal.pone.0097441)
Supplement: Table S1 — Archaea encoding partial rmlABCD clusters. (DOC) [file pone.0097441.s002.doc]

**Table S1 – Archaea encoding partial *rmlABCD* clusters**

| **Species** | **RmlA** | **RmlB** | **RmlC** | **RmlD** |
| --- | --- | --- | --- | --- |
|  |  |  |  |  |
| *Ferroglobus placidus* | Ferp_2391 |  | Ferp_2401 | Ferp_2397 |
| *Metallosphaera sedula* | Msed_1845 | Msed_1850 | Msed_1851 |  |
| *Methanobacterium* sp. AL-21 | Metbo_0919 |  |  | Metbo_0920 |
| *Methanobrevibacter ruminantium** | mru_1062 | mru_1060 | mru_1061 |  |
| *Methanofollis liminatans* | Metli_1368 | Metli_1369 |  |  |
| *Methanosarcina acetivorans** | MA2183 | MA2186 |  | MA2184 |
| *Methanosarcina mazei* |  | MmTuc01_1219 | MmTuc01_1218 | MmTuc01_1220 |
| *Methanothermococcus okinawensis* | Metok_1466 | Metok_1465 |  | Metok_1464 |
| *Pyrobaculum* sp. 1860 | P186_1959 |  | P186_1956 | P186_1957 |
| *Sulfolobus islandicus* HVE10/4 | SiH_0444 | SiH_0443 |  | SiH_0445 |
| *Sulfolobus islandicus* L.D.8.5 | LD85_0642 | LD85_0641 |  | LD85_0643 |
| *Sulfolobus islandicus* L.S.2.15 | LS215_1052 | LS215_1053 |  | LS215_1051 |
| *Sulfolobus islandicus* M.14.25 | M1425_0608 | M1425_0607 |  | M1425_0609 |
|  | M1425_0978 | M1425_0977 |  | M1425_0979 |
| *Sulfolobus islandicus* M.16.27 | M1627_0617 | M1627_0616 |  | M1627_0618 |
|  | M1627_1031 | M1627_1030 |  | M1627_1032 |
| *Sulfolobus islandicus* M.16.4 | M164_0635 | M164_0634 |  | M164_0636 |
| *Sulfolobus islandicus* Y.G.57.14* | YG5714_0572 | YG5714_0571 |  | YG5714_0573 |
| *Sulfolobus islandicus* Y.N.15.51 | YN1551_2445 | YN1551_2446 | YN1551_2443 |  |
|  | YN1551_2294 | YN1551_2293 |  | YN1551_2295 |
| *Sulfolobus solfataricus* P2* | SSO1782 | SSO1781 |  | SSO1783 |
| *Vulcanisaeta moutnovskia* | VMUT_0078 | VMUT_0077 |  |  |
|  |  |  |  |  |

^*^ A complete *rmlABCD* cluster is also encoded by this species
